# ﻿Morphometric parameters of seeds as a practical method for identifying rare species of the genus *Tulipa* L. (Liliaceae) from East Kazakhstan region

**DOI:** 10.3897/phytokeys.251.133890

**Published:** 2025-01-16

**Authors:** Aidar A. Sumbembayev, Olga Lagus, Alevtina N. Danilova, Agnieszka Rewicz, Sławomir Nowak

**Affiliations:** 1 Altai Botanical Garden, Ermakov St., Ridder 071300, Kazakhstan Altai Botanical Garden Ridder Kazakhstan; 2 Department of Geobotany and Plant Ecology, Faculty of Biology and Environmental Protection, University of Lodz, Banacha 12/16, 90-237 Lodz, Poland University of Lodz Lodz Poland; 3 Department of Plant Taxonomy and Nature Conservation, Faculty of Biology, University of Gdansk, Wita Stwosza 59, 80-308 Gdańsk, Poland University of Gdansk Gdańsk Poland

**Keywords:** 3D ultrastructure, ecological factors, Kazakh Altai, morphometric characteristics, Scanning Electron Microscope (SEM), seed coat, seed morphology, taxonomy, *Tulipa*

## Abstract

The genus *Tulipa* includes some of the most important ornamental plants. The aim of this work was to study the seed morphology of *Tulipa* species from East Kazakhstan, including seed coat structure. An analysis focused on five taxa from various natural environmental conditions. A total of 31 tulip populations were studied to establish morphological variability. Preliminary analyses of the importance of habitat-related ecological factors have been carried out. The results of this study provide new qualitative characteristics for distinguishing closely related species and are discussed in relation to their systematic relationships. The structure of the seed coat was studied by Scanning Electron Microscopy; however, the results did not show significant variability. An identification key to determine the species of tulips in East Kazakhstan is proposed.

## ﻿Introduction

The genus *Tulipa* L. is one of the largest in the family Liliaceae, whose range extends enormously from Portugal and the northern regions of Africa across the entire Eurasian continent to the southern islands of Japan (Zonneveld 2009). All representatives of the genus are known as rare, highly ornamental plants. In terms of color variation, originality of shape, beauty and flower size, many species of wild tulips have a long history in cultivation (Christenhusz et al. 2013; Wilson 2022). However, wild tulips at the present stage are subject to strong anthropogenic pressure and without strengthening conservation efforts, the future of these beautiful plants in Central Asia may be in serious danger (Wilson et al. 2021; Dekhkonov et al. 2023). Taking into account limiting factors such as the narrow ecological amplitude of the species and anthropogenic influence, many species of tulips, including *Tulipabiflora* Pall., *T.patens* C.Agardh, *T.uniflora* (L.) Bess. ex Baker, *T.heteropetala* Ledeb., growing in the territory of East Kazakhstan region, are included in the Red Book of Kazakhstan (Red Book of Kazakhstan 2014), and *T.altaica* Pall. has a protected status in the adjacent region, the Altai region (Russia) (Alexandrova et al. 2006).

The genus *Tulipa* has 94 generally known species (POWO 2024), of which 42 species are reported for Kazakhstan (Ivashchenko and Belyalov 2019; Wilson 2022), representing three subgenera: *Orythia*, *Tulipa* and *Eriostemones* (Christenhusz et al. 2013). The study of wild Kazakh tulips began only at the end of the 18^th^ century. According to Kotukhov (2005), there are five species within the Kazakh Altai, Saur-Manrak and Zaisan Basins (East Kazakhstan region): *T.patens* C.Agardh, *T.altaica* Pall. ex Spreng., *T.biflora* Pall., *T.uniflora* (L.) Bess. ex Baker, *T.heteropetala* Ledeb. These species are protected in Kazakhstan, adjacent regions (East Kazakhstan region), the Altai region (Russia) (Alexandrova et al. 2006) and China (Qin et al. 2017), as well as in the IUCN Red Book (IUCN 2024) (Table 1).

**Table 1.** Rarity status of tulip species from East Kazakhstan.

Species	Status of the species in the Red Book of Kazakhstan	Status of the species in the Red Book of the Altai region (adjacent region)	Status of the species in the Red Book of China (adjacent region)	Status of the species in the IUCN
*Tulipapatens* C.Agardh	III category. Rare. Species decreasing in numbers	R(b) – Rare. Rare species found in few localities	–	LC – least concern
*T.altaica* Pall. ex Spreng.	–	V(b) – Vulnerable. Vulnerable species, the northeastern border of its range passes in the region	–	LC – least concern
*T.biflora* Pall.	I category. Rare. Threatened	–	–	LC – least concern
*T.uniflora* (L.) Bess. ex Baker	III category. Rare. Species with decreasing range	V(b) – Vulnerable. Vulnerable species. The northern border of its range passes through the region.	VU – Vulnerable	NT – near threatened
*T.heteropetala* Ledeb.	II category. Species is a native to Altai, occurring in small numbers.	V(a) – Vulnerable. Vulnerable species. Altai-Sayan endemic	–	LC – least concern

The aforementioned threats, including strong anthropopressure, both on habitats and uncontrolled plant harvesting, make easy identification of species at different stages of development extremely important for conservation purposes.

There are numerous studies on the biology of the genus *Tulipa* in terms of taxonomy (Mordak 1990; Christenhusz et al. 2013; Wilson 2022), ontogenesis (Abdusalam and Tan 2014), genetics (Zonneveld 2009; Kiani et al. 2012; Pourkhaloee et al. 2018; Asgari et al. 2020; Haerinasab et al. 2021; Hajdari et al. 2021; Li et al. 2021), population geography (Tojibaev and Beshko 2015; Trias-Blasi et al. 2017; Abduraimov et al. 2018; Pechenitsyn et al. 2020; Dekhkonov et al. 2023) and karyology (Masoud et al. 2002). However, studies on the structure and variability of seeds in the genus are still lacking. There are a limited number of works on the seed productivity of tulips (Zhang et al. 2020; Lidzhieva et al. 2021; Zhang et al. 2023).

The study of the morphology of seeds of closely related species is highly important in terms of biodiversity. The morphology of seeds, along with the morphology of flowers, inflorescences and fruits, is among the dominant generative characteristics of taxonomy (Dekhkonov et al. 2022). The knowledge gained helps to find the place of species in genera and families, as well as in general plant classification. The external structure of seeds, surface structure features, and weight characteristics can serve as important additional features in the taxonomy of the genus.

Understanding both the macro- and microstructure of seeds is highly important for plant taxonomy and phylogenetic inference, as highlighted by various studies (Duckett and Soni 1972a, 1972b; Behnke and Barthlott 1983; Mourad et al. 2010). The application of biometric and seed sculpture analysis has proven to be a valuable tool for tasks such as classification, ecological studies, and identification of species. Seed characteristics play crucial roles in linking phenotypic features to generic relationships and in circumscribing subtribes within various genera (Heywood and Dakshini 1971; Pfosser et al. 2003; Rewicz et al. 2017). Seed shape, in particular, has emerged as a vital aspect in certain taxonomic studies, standing alongside seed sculpture as one of the most consistent species-specific traits (Pfosser et al. 2003).

The morphology of generative organs, particularly seeds, could be a key feature in determining the species identity of a plant (Lipińska et al. 2022; Sumbembayev et al. 2023). Moreover, data on morphology for many species of the *Tulipa* genus are incomplete (Zonneveld 2015; Xing et al. 2017; Dekhkonov et al. 2022), and for Kazakhstan, they are fragmentary (Zonneveld and De Groot 2012; Epictetov and Belyalov 2013; Ivashchenko and Belyalov 2019).

The purpose of this study was to assess the morphological parameters for seeds of the *Tulipa* species from the East Kazakhstan region (based on biometric traits and scanning electron microscopy), as well as to analyze their biometric according to the ecological and habitat factors in the study region, including systematic relationships in the genus. Finally, to propose a dichotomous key to species determination based on the analyzed features.

## ﻿Materials and methods

### ﻿Biometric analyses

**Figure 1.**
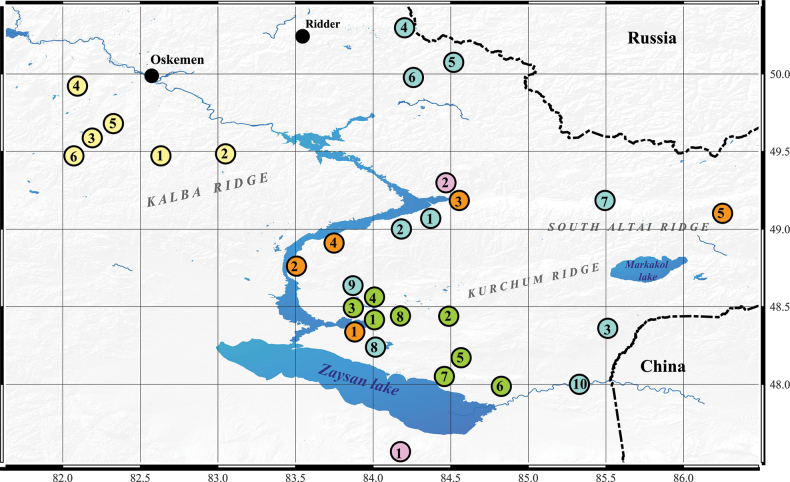
Collection sites of studied species of the genus *Tulipa* in the East Kazakhstan region. The color of the dot reflects the species: yellow - *T.uniflora*, orange - *T.patens*, pink - *T.biflora*, green - *T.altaica*, turquoise - *T.heteropetala*, and the numbers reflect the populations studied.

The focus of this study was the seeds of five species (*T.altaica*, *T.biflora*, *T.heteropetala*, *T.patens*, *T.uniflora*) of tulips from 31 populations collected from natural populations in the East Kazakhstan region in various ecological and geographical habitats (Fig. 1). The species nomenclature is based on POWO (2024). The systematics of the genus and sectional division are accepted according to the works of Van Raamsdonk et al. (1996), Christenhusz et al. (2013), and Wilson (2022).

The survey territory was the entire East Kazakhstan region. It has an area of more than 97 thousand square kilometers, which borders the Altai region (Russia) in the north and China in the west (Fig. 1). The region has a wide range of natural conditions, from deserts to alpine meadows.

Seed material was collected in sites from East Kazakhstan region. Collections were made from 2010–2020 by Professor Yu.A. Kotukhov; the seeds were stored dry in paper bags at 18–20 °C. Fully formed seed samples were selected for the study. All thirty-one studied populations are characterized in details by geographical coordinates, description of the locality and habitat, and the Ellenberg indicators values in the Suppl. material 1.

**Figure 2.**
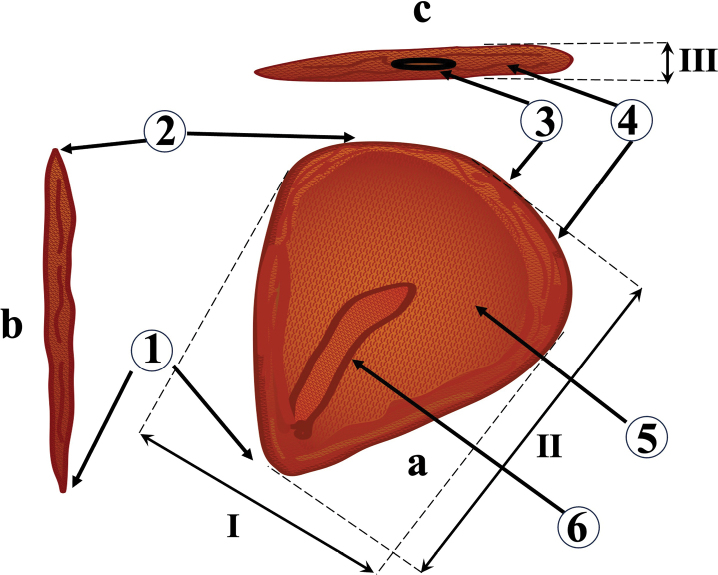
Schematic seed structure of the genus *Tulipa*: **a** main view **b** side view **c** top view. Seed features are marked by numbers (with Latin terms in brackets): 1 – micropyle (*micropyle*), 2 – chalazal end (*chalaza*), 3 – seed hilum (*hilum*), 4 – seed suture (*raphe*), 5 – endosperm (*endospermium*), and 6 – embryo (*embryo*). Measurements taken in this study: I – width, II – length, and III – thickness.

Twenty seeds from each population of *Tulipa* were analyzed. The following four seed traits were quantified: a) length, b) width, c) thickness, and d) weight of 1000 seeds. Moreover, the qualitative characteristics of the seeds, such as color, shape, testa surface, state of the micropyle (***micropyle***), chalazal end (***chalaza***), seed hilum (***hilum***), structure and surface of the seed suture (***raphe***), endosperm (***endospermium***) and embryo (***embryo***), were described (Fig. 2).

### ﻿SEM analyses

The seeds were sputter-coated with a 4 nm layer of gold before being subjected to SEM observation. Seed ornamentation characteristics were studied by Scanning Electron Microscopy (SEM) (Phenom Pro X) at the Department of Invertebrate Zoology and Hydrobiology, University of Lodz (Poland) (Suppl. material 4: figs S1–S5). Seed surface ultrastructure 3D models were generated via 3D roughness reconstruction via a phenom electron microscope (Fig. 4). The digital images obtained via SEM were trimmed and arranged in plates via Corel Draw 2018.

The shapes of individual seed coat cells, the anticlinal wall, and the surface structure of the periclinal wall are classified into Barthlott (1984).

### ﻿Statistical analyses

Seed morphology was studied via a MAGUS D9T stereomicroscope. The shape and surface of the seeds were described using the method proposed by Martin (1946). Schemes of seed structure based on average metric characteristics were designed in the AutoCAD graphic program. The diagrams show the appearance of the studied seeds. The maximum seed sizes are indicated by a dotted line.

The Shapiro–Wilk and Kolmogorov–Smirnov tests were conducted to check for a normal distribution of the data. One-way analysis of variance (ANOVA) with statistics F was used to determine whether the investigated morphological seed traits differed among the *Tulipa* populations (Zar 1984). Cluster analysis UPGMA tree was constructed using R (R Core Team 2021) with packages: poppr (Kamvar et al. 2014), ggplot2 (Wickham 2016), dplyr (Wickham et al. 2023), dendextend (Galili 2015), RColorBrewer (Neuwirth 2014), vegan (Oksanen et al. 2017), GUniFrac (Chen et al. 2023), labdsv (Roberts 2023).

Principal component analysis (PCA), correlation analysis, ANOVA and species dendrograms were constructed in R (with packages; dplyr, dendextend, RColorBrewer, ggplot2, vegan, GUniFrac, labdsv). The ecological conditions of the habitats of the studied populations were assessed according to the Ellenberg ecological scales (Tichý et al. 2023). Seed color was established according to the MacAdam (1974) color scale.

## ﻿Results

**Figure 3.**
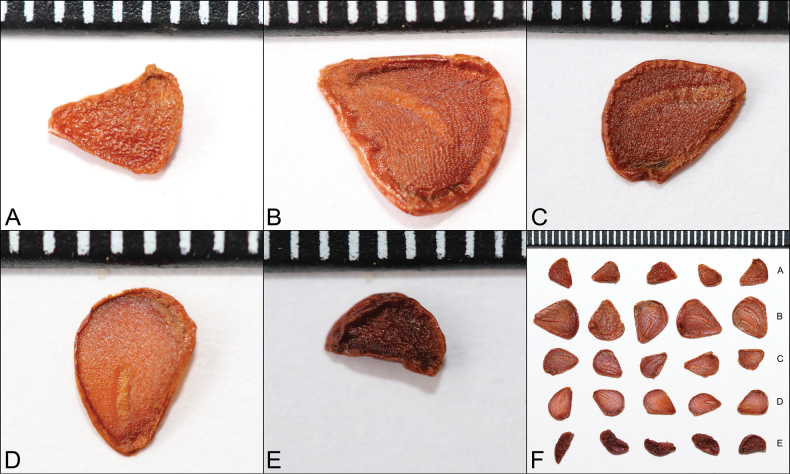
Seeds of the genus *Tulipa*: **A***T.heteropetala***B***T.altaica***C***T.biflora***D***T.patens***E***T.uniflora***F** comparison of studied seeds of the genus *Tulipa* of the Kazakh Altai.

In the study area, East Kazakhstan, the largest populations of tulips were found in the Zaisan depression, as well as the Narym and Kurchum ridges. The occurrence is dominated by rocky sites and diverse vegetation steppes, as well as scrub (Suppl. material 1). Examination of the seed morphology of five species of tulips collected under different ecological and geographic conditions showed similarity within species but variation in shape and size among them (Fig. 3).

**Figure 4.**
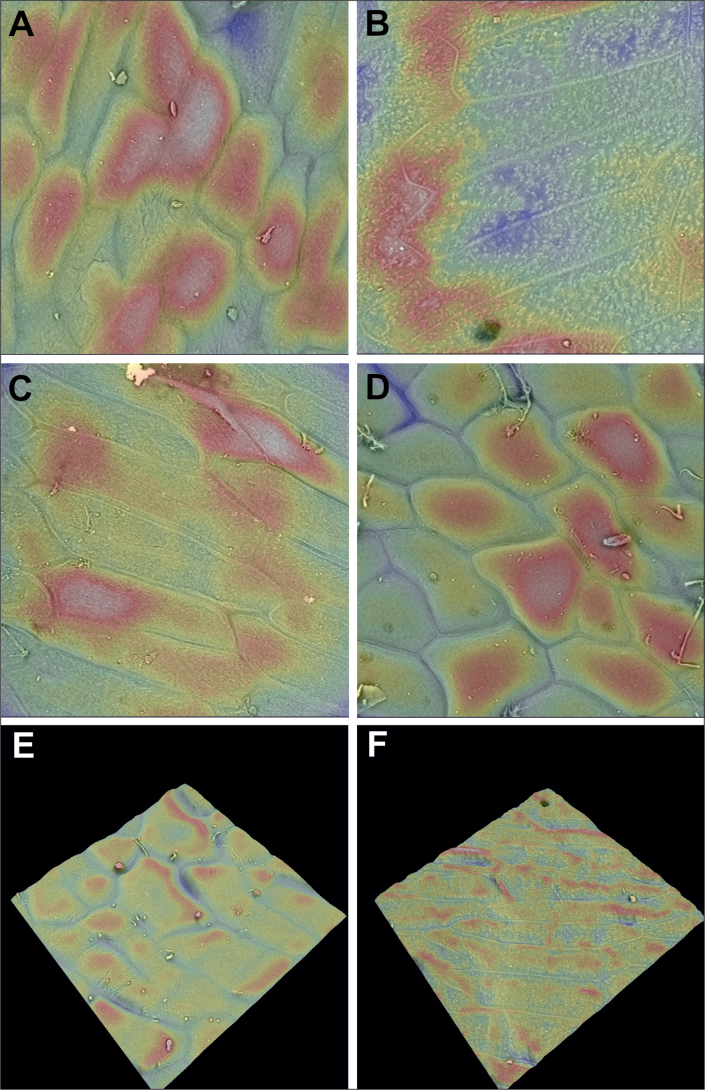
Three-dimensional models of the ultrastructure of the surfaces of the analyzed seeds: **A***T.heteropetala***B***T.uniflora***C***T.altaica***D, E***T.biflora***F***T.patens*.

The study of the external morphometry of tulip seeds, which is based on linear dimensions (length, width and thickness), as well as the weight of the seeds, clearly revealed a high degree of variation in all the parameters (Suppl. material 2).

A description of the external and internal structure of *T.patens*, *T.altaica*, *T.biflora*, *T.uniflora*, and *T.heteropetala* made it possible to identify morphological features characterizing the external and internal characteristics of tulip seeds for practical use in species determination and with reference to the systematic relationships (Suppl. material 3).

The color of the seeds of the studied species of the genus *Tulipa* ranges from brown to reddish-brown. The shape of the seeds is flat, vaguely triangular or wedge-shaped and curved wedge shaped. The test surface varies from rough to wrinkled. In all the studied species, the micropyle is overgrown, the seed hilum is depressed and longitudinally slit-like, the raphe from thickened and smooth to thickened, the endosperm is developed, and the shape of the embryo is either straight or curved toward the raphe or strongly curved.

*T.altaica* produced the largest and heaviest seeds. The width of the seeds of *T.uniflora* was the smallest, while the weight of the 1000 seeds differed slightly from those in *T.altaica*. Compared with *T.altaica*, the seeds of *T.heteropetala* should be classified as small. *T.patens* has the lightest and flattest seeds. The average length and weight of 1000 seeds of *T.biflora* and *T.uniflora* differ slightly from each other; significant differences in these species were found in the width and thickness of the seeds.

Analysis of seed micromorphology via SEM and 3D models (Fig. 4) did not reveal significant differences in the structure of the seed coat. The anticlinal walls in all the species are straight, and the periclinal walls are mostly smooth (e.g., Suppl. material 4: figs S1A–D, S2C, D, S3B). Analyses of the shape of individual seed coat cells revealed that, in most species, they are elongated and rectangular (e.g. Suppl. material 4: figs S1B, S2C) or elongated and rounded (e.g. Suppl. material 4: figs S4B, S5A). More or less irregular or rectangular cells were observed on the edges of the seeds (e.g., Suppl. material 4: figs S1A, S3F, S5C).

**Figure 5.**
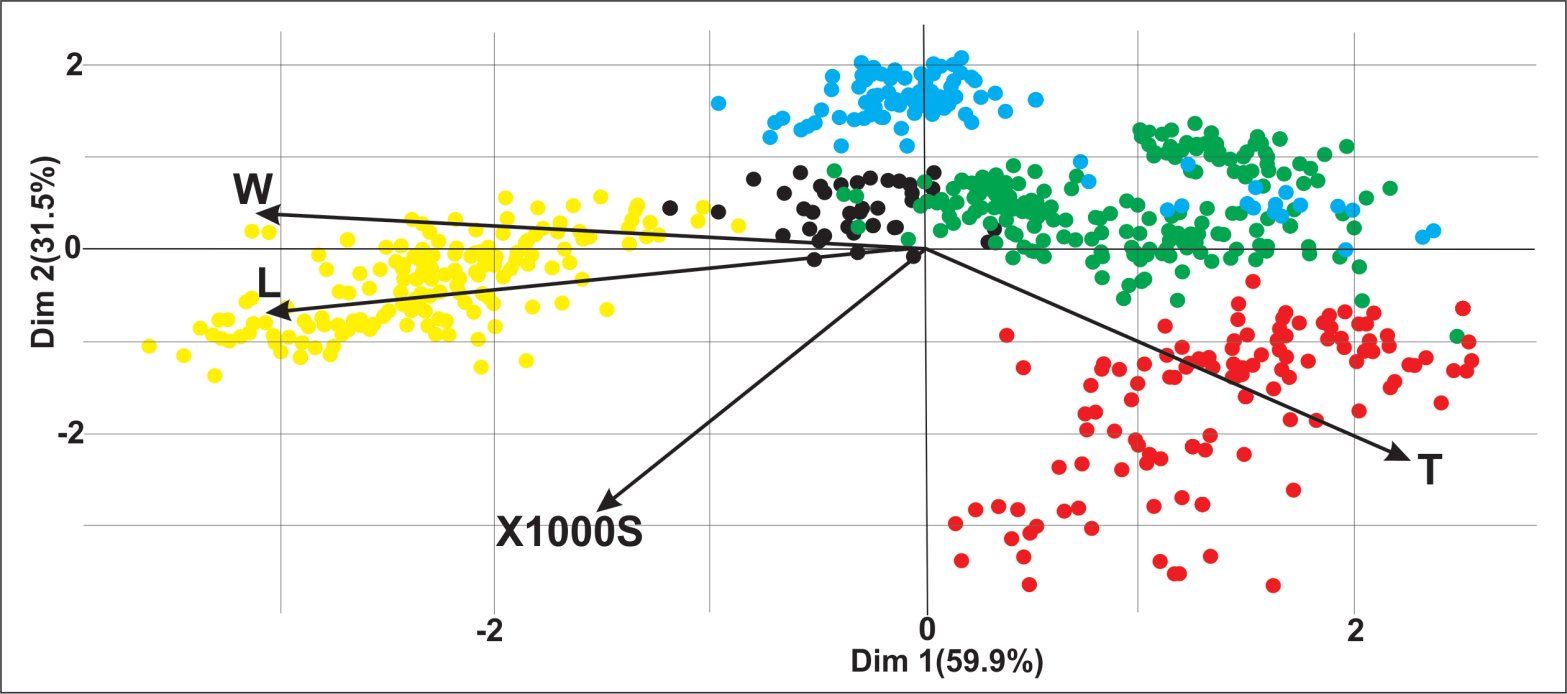
Principal component analysis (PCA) of individual seeds measured of the genus *Tulipa* in East Kazakhstan. Explanation: W – width of seed, L – length of seed, T – thickness of seed, X1000S – weight of 1000 seeds. Each dot represents one individual seed measured and the color of the dot reflects the species: yellow - *T.altaica*, black - *T.biflora*, green - *T.heteropetala*, blue - *T.patens*, red - *T.uniflora*.

Principal component analysis (PCA) of individuals of the genus *Tulipa* (Fig. 5) revealed the quality of the samples performed and demonstrated the differences and similarities among all the samples of the studied species. Significant isolation of *T.altaica* samples from other species along coordinate 1 was revealed. *T.uniflora* and *T.patens* are qualitatively separated from other species along coordinate 2. All the species are qualitatively separated from each other, with almost no overlaps. Species of the subgenus *EriostemonesT.biflora* and *T.patens* are significantly separated along coordinate 2 and are not intersected by ellipses. Species of the section Orithyia: *T.uniflora* and *T.heteropetala* are located quite close together, but there are practically no noticeable intersections, and these species are qualitatively separated along coordinate 2, which indicates the independence of the species under consideration.

**Figure 6.**
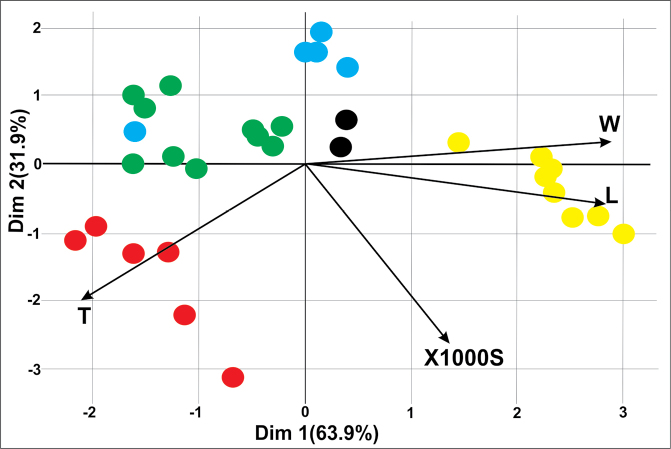
Principal component analysis (PCA) for populations of species of the genus *Tulipa* from East Kazakhstan. Explanation: W – width of seed, L – length of seed, T – thickness of seed, X1000S – weight of 1000 seeds. Each dot represents one population studied and the color of the dot reflects the species: yellow - *T.altaica*, black - *T.biflora*, green - *T.heteropetala*, blue - *T.patens*, red - *T.uniflora*.

Principal component analysis (PCA) of species differences (Fig. 6) revealed similarities and differences between populations within each tulip species. A clear isolation of *T.altaica* populations from populations of other species was revealed. The populations of *T.heteropetala* and *T.uniflora* are spatially separate. Remarkably, the narrowness of the population distribution of these species indicates that the choice of the number of populations for analysis is sufficient.

Cluster analysis of the ranking of populations of species of the genus *Tulipa* according to external morphometric and weight characteristics, presented in a dendrogram (Suppl. material 5), graphically arranged the studied populations according to similarity and difference. All *T.altaica* populations were included in cluster 1. High similarity of seed material was established between populations of the species *T.heteropetala* and *T.patens*, forming cluster 2. Importantly, the populations of *T.heteropetala* and *T.uniflora*, which belong to the same section, *Orithyia*, are located strictly in different clusters—in the second and third clusters, respectively.

**Figure 7.**
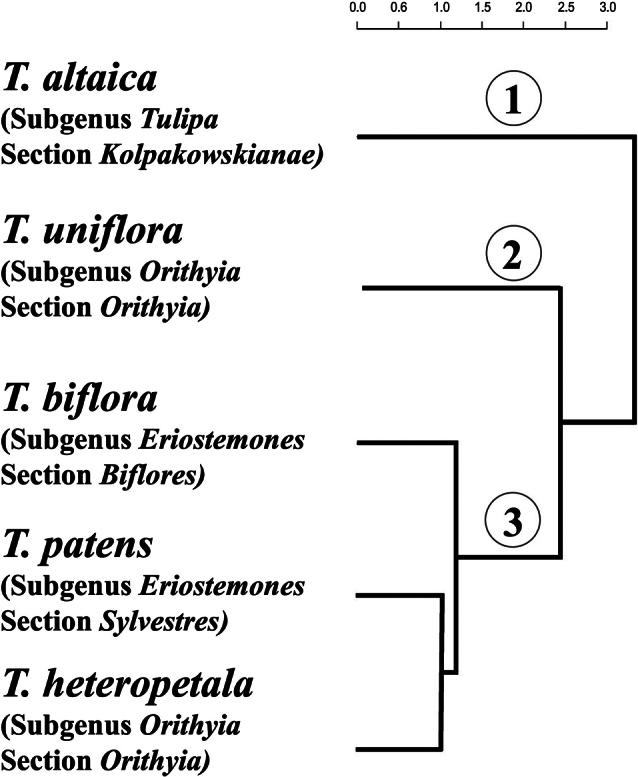
Dendrogram of the interspecific similarity of species of the genus *Tulipa* from East Kazakhstan.

The cluster dendrogram (Fig. 7), which was constructed on the basis of interspecific similarity and differences, revealed the morphological consistency of the seed material in *T.patens*, *T.biflora* and *T.heteropetala*, which formed single cluster 3. Further, species belonging to the same section Orithyia, *T.uniflora* and *T.heteropetala*, are located in different clusters.

Correlation analysis of the dependence of the main linear and weight characteristics of seeds on environmental conditions revealed strong, stable direct and inverse correlations in all species of the genus *Tulipa* (Suppl. material 6). Strong direct correlations were established for *T.patens* and *T.uniflora* between the weight of 1000 seeds and soil moisture (M) and nutrients (N). A weak inverse correlation was also observed between seed length and soil salinity in *T.patens* and *T.altaica*.

Analysis of variance (ANOVA) (Table 2) revealed that some environmental factors can significantly influence the morphometric characteristics of seeds of the genus *Tulipa*. The following significant influences of environmental factors were established: temperature (T) on the morphometric characteristics of *T.heteropetala*, *T.patens* and *T.uniflora*; light (L) on the morphometric characteristics of *T.patens* and *T.uniflora*; and nutrients (N) for *T.patens*, *T.heteropetala*, and *T.uniflora*.

**Table 2.** Results of analysis of variance (ANOVA) by species.

	Factor/trait	L	T	M	R	N	S	R:N	R:S	L:T	T:M	T:R
*T.altaica*	length				***	***	***					
	width		.			*	***	**	***			
	thickness				**	**						
	weight of 1000 seeds		***	***	***	***	***	***	***			
*T.biflora*	length	.	.	.								
	width	**	**	**								
	thickness	***	***	***								
	weight of 1000 seeds	***	***	***								
*T.heteropetala*	length		***	**		***	***			***	***	.
	width		***			***	***			***	***	
	thickness		***	***		***	**			***	***	***
	weight of 1000 seeds	***	***	***	***	***	***			***	***	***
*T.patens*	length	***	***			***						
	width	**										
	thickness	***	***	***		***						
	weight of 1000 seeds	***	***	***		***						
*T.uniflora*	length	***	**	***		***						
	width	***		***		***						
	thickness	**	*	***		**						
	weight of 1000 seeds	***	***	***		***						

Significance codes: 0 ‘***’ 0.001 ‘**’ 0.01 ‘*’ 0.05 ‘.’ 0.1 ‘ ’ 1.

ANOVA (Table 3) for the all studied *Tulipa* species from East Kazakhstan combined, revealed a significant effect of almost all the environmental factors on seed length. In addition, the size and weight of the seeds are influenced the most by light (L) and nutrients (N).

**Table 3.** Results of analysis of variance (ANOVA) by genus.

Factor/trait	Species	L	T	M	R	N	S	species:L	species:T	L:T	species:M	T:M	species:R	T:R	M:R	species:N	R:N
length	***	**	**	***	***	***		***	***	**	***	*	***	.		***	***
width	***	*	**	***		***		***	.	**	***		***			***	
thickness	***	***		.		***		***	***	***	***	.	*	***		***	
weight of 1000 seeds	***	*	.	***		***		***	***	*	***	***	***	***	***	***	***

Significance codes: 0 ‘***’ 0.001 ‘**’ 0.01 ‘*’ 0.05 ‘.’ 0.1 ‘ ’ 1.

**Figure 8.**
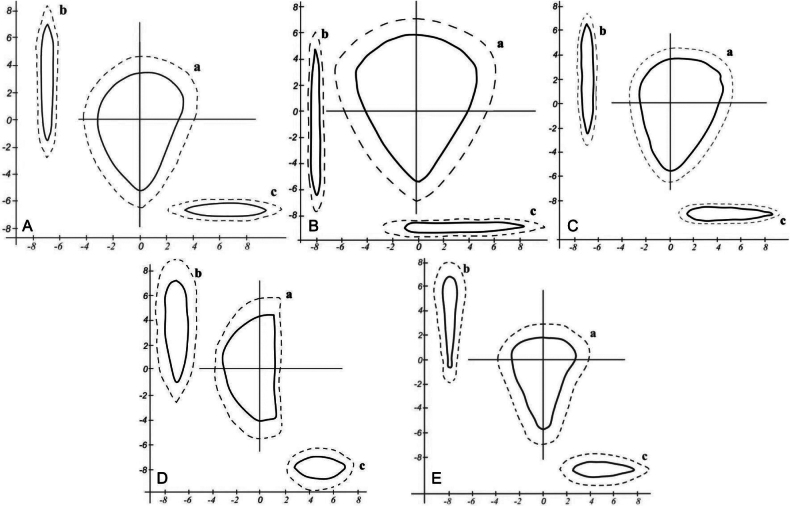
Schemes of the standard external structure of species of the genus *Tulipa* from the East Kazakhstan region, compiled on the basis of average linear sizes: **A***T.patens***B***T.altaica***C***T.biflora***D***T.uniflora***E***T.heteropetala*. a – main view; b – side view; c – top view; maximum dimensions are indicated by a dotted line.

Summarizing morphometric analysis for populations of the *Tulipa* species, diagrams of the standard external structure of tulip seeds from the East Kazakhstan region were constructed (Fig. 8).

## ﻿Discussion

The study of rare and endangered plant species is one of the main tasks in solving the problem of preserving the biological diversity of Kazakhstan. Since monitoring nature is largely based on morphology, providing more data in helping their accurate identification should be the most accessible and reliable as possible. The study of similar species of tulips is actively carried out throughout the range of these species. Previously, Kiani et al. (2012) and Asgari et al. (2020) carried out molecular analyses of *T.biflora* and other tulips in Iran. Li et al. (2021) performed phylogenetic analysis of *T.patens* and *T.altaica* in China. Masoud et al. (2002) performed a karyological study of *T.biflora* and other tulip species in Iran. Xing et al. (2017) studied the morphology of *T.altaica* and other wild tulip species in Xinjiang (Western China). Tojibaev and Beshko (2015) studied the distribution and status of populations of *T.biflora* and other tulips in Uzbekistan.

As a result of our study, the morphobiological characteristics of seeds for five species of the genus *Tulipa* in the East Kazakhstan region were determined. Thus, our results complement the fundamental data on the external and internal structure of species such as *T.patens*, *T.altaica*, *T.biflora*, *T.uniflora*, and *T.heteropetala* and have a great potential to be used for species identification in a region.

The sizes of the seeds of the studied species differ slightly, which indicates moderate heterogeneity of the seeds and low adaptive potential of the studied plants. This fact indicates a narrow range of *Tulipapatens*, *T.altaica*, *T.biflora*, *T.uniflora*, and *T.heteropetala* in the regional flora. A comparison of the weight indicators of species of the genus *Tulipa* also revealed slight variability in this trait across the region. Principal component analysis (PCA) for populations and for individuals of species of the genus *Tulipa* reliably and qualitatively separates all species at both the species and section levels. Populations of *T.altaica* are significantly different from those of other tulips in East Kazakhstan. However, a limiting element of our study is the lack of embryo measurements, and it is worth conducting such studies for the genus *Tulipa* in the future. Recent studies indicate (Carta et al. 2024) that this may be one of the key traits in the analysis of seed functionality and the evolutionary trends in angiosperms.

Cluster analysis and a dendrogram for interspecific similarity of species graphically demonstrated the clear distinctiveness of *T.altaica* and *T.uniflora* and the lack of such for *T.heteropetala* and *T.patens*.

Correlation analysis of the dependence of seed size and weight on growing conditions indicate the potential dependence on soil moisture and richness, as well as soil salinity. In addition, no constant relationship has been established between seed size and important factors for flowering plants, such as light and temperature. ANOVA revealed that environmental factors had a greater influence on seed length, and, the richness of the soil affect the seed size. However, the results obtained should be treated as preliminary research due to the limitation of the samples collected. The species studied show a much wider range of occurrence than within the East Kazakhstan region, so a more comprehensive study requires collecting a representative sample from the entire distribution range and including climatic data. Especially since studies show (Carta et al. 2022) that climate is a strong predictor of germination response, moreover, this trait has a strong association with phylogeny.

Importantly, qualitative differences in the external and internal characteristics of the seeds of two species, *T.uniflora* and *T.heteropetala*, were observed during the study. The characteristics of the seeds of these two species are qualitative features for distinguishing them as independent species. On the basis of our research, we consider that distinctiveness of *T.heteropetala* could be justified.

The study of seed coat micromorphology did not reveal significant differences between the examined *Tulipa* species. These differences are minimal or insignificant, making it difficult to distinguish species on the basis of seeds. In the context of the studied species, the lack of differentiation suggests that seed micromorphology is not a useful tool for their identification (Heywood and Dakshini 1971). There are several potential reasons for this phenomenon. First, the sculpture of the seed coat may be too conservative, meaning that it has not undergone significant evolutionary changes among different species. Such conservatism may result from a stable environment that does not exert selective pressure, leading to differentiation of the seed ultrastructure. Additionally, variability of seed structure could be very limited due to the strong conservatism of the features related to their optimal adaptation to fulfill their primary functions (Stace 1993).

The phylogeny within the genus *Tulipa* is rather well understood, and many relationships are resolved. However, many species concepts still need to be clarified. Within the studied species, for example, there are different definitions of *T.altaica* or the complexity of *T.biflora* (Wilson 2022; Sutula et al. 2024). It seems that phylogenetic relationships do not have much significance in the case of seed structure, so many similar traits can develop under environmental pressures, which may suggest the statistical significance of some of the factors studied (Tables 2, 3; Suppl. material 6). Closely related and classified within one section, species such as *T.uniflora* and *T.heteropetala* do not show much similarity (Wilson 2022; Sutula et al. 2024). On the other hand, *T.uniflora* was the only one to be collected from a distinct area, which may confirm that the impact of habitat factors is important in shaping seed traits in the genus *Tulipa*.

## ﻿Conclusion

The genus *Tulipa* in East Kazakhstan is represented by five rare and endangered species: *T.patens*, *T.altaica*, *T.biflora*, *T.uniflora* and *T.heteropetala*. The seeds of all species of tulips from East Kazakhstan differ qualitatively from each other not only in size and weight but also morphologically in characteristics such as micropyle, chalazal end, seed hilum, raphe, endosperm and embryo.

As a result of this study, the possibility of using the morphology of tulip seeds as a systematic feature was confirmed. The difference in seed material was clearly shown in two closely related species from the *Orithyia* section: *T.uniflora* and *T.heteropetala*. Currently, the morphology of seeds is the most reliable feature for distinguishing these closely related species. The study of the comparative morphological characteristics of tulip seeds has made it possible to identify a group of characteristics that can be used to clarify the taxonomic affiliation of species. The established morphological characteristics of the seeds made it possible to develop an identification key for species of the genus *Tulipa* in the East Kazakhstan region. However, it is impossible to identify a single seed trait that differentiates all species. Therefore, the determination key uses only the key features and the best discriminating species. To obtain the best possible determination, we suggest using a set of various data, including descriptive ones as in this research.

On the other hand, the ultrastructures of seeds do not show much variation, most likely because the high conservatism of seed structure and phylogenetic relationships are not reflected in the seed structure. However, owing to the demonstration of overall seed variability, it is worthwhile to undertake research on a larger sample of the genus *Tulipa* to fully understand the reasons for this variability. This is especially true for a genus of great ornamental importance that is highly under threat.

### ﻿Key to the *Tulipa* species in the East Kazakhstan region

**Table:** 

1	Seeds are noticeably thickened in the basal part and curved in shape; the tips of the seeds and the chalaza are turned toward the raphe; the testa is longitudinally furrowed; the chalazal end is transversely grooved	***T.uniflora* (L.) Bess. ex Baker.**
–	Seeds with other characteristics	**2**
2	The seeds are quite large, up to 7 mm in length and around 6 mm in width, with a noticeably elongated tip; the surface of the seeds is finely veined; the chalazal end is smooth	***T.altaica* Pall. ex Spreng.**
–	The seeds are small, without an elongated tip; chalazal end is rough (tubercle-wrinkled)	**3**
3	The seed surface is finely tuberous; the seed embryo is strongly curved toward the raphe	***T.biflora* Pall.**
–	The seed surface is rough and wrinkled; the embryo is rectilinear in shape	**4**
4	The seeds are brown; the seed hilum is protruding and longitudinally slit-shaped; and the raphe has a smooth surface	***T.patens* C.Agardh**
–	Seeds are orange-brown to reddish-brown in color; the seed hilum is concave and ellipsoidal in shape; raphe is grooved	***T.heteropetala* Ledeb.**

## ﻿Additional information

### Author ORCIDs

Aidar A. Sumbembayev https://orcid.org/0000-0003-0682-9162

Olga Lagus https://orcid.org/0000-0001-7178-2888

Alevtina N. Danilova https://orcid.org/0000-0002-1096-9339

Agnieszka Rewicz https://orcid.org/0000-0002-8228-6406

Sławomir Nowak https://orcid.org/0000-0002-0496-1023

### Data availability

All of the data that support the findings of this study are available in the main text or Supplementary Information.

## Supplementary materials

Supplementary material 1 Description of the population locations of *Tulipa* species in East Kazakhstan region Data type pdf https://binary.pensoft.net/file/1221440This dataset is made available under the Open Database License (http://opendatacommons.org/licenses/odbl/1.0/). The Open Database License (ODbL) is a license agreement intended to allow users to freely share, modify, and use this Dataset while maintaining this same freedom for others, provided that the original source and author(s) are credited.Aidar A. Sumbembayev, Olga Lagus, Alevtina N. Danilova, Agnieszka Rewicz, Sławomir Nowak

Supplementary material 2 Morphometric characteristics of *Tulipa* seeds from different growing areas Data type pdf https://binary.pensoft.net/file/1221441This dataset is made available under the Open Database License (http://opendatacommons.org/licenses/odbl/1.0/). The Open Database License (ODbL) is a license agreement intended to allow users to freely share, modify, and use this Dataset while maintaining this same freedom for others, provided that the original source and author(s) are credited.Aidar A. Sumbembayev, Olga Lagus, Alevtina N. Danilova, Agnieszka Rewicz, Sławomir Nowak

Supplementary material 3 Indicators of morphological characteristics of seeds of species of the genus *Tulipa* in East Kazakhstan region Data type pdf https://binary.pensoft.net/file/1221442This dataset is made available under the Open Database License (http://opendatacommons.org/licenses/odbl/1.0/). The Open Database License (ODbL) is a license agreement intended to allow users to freely share, modify, and use this Dataset while maintaining this same freedom for others, provided that the original source and author(s) are credited.Aidar A. Sumbembayev, Olga Lagus, Alevtina N. Danilova, Agnieszka Rewicz, Sławomir Nowak

Supplementary material 4 Seed coat micromorphology of *Tulipa* species, seen under Scaning Electron Microscope Data type pdf https://binary.pensoft.net/file/1221443This dataset is made available under the Open Database License (http://opendatacommons.org/licenses/odbl/1.0/). The Open Database License (ODbL) is a license agreement intended to allow users to freely share, modify, and use this Dataset while maintaining this same freedom for others, provided that the original source and author(s) are credited.Aidar A. Sumbembayev, Olga Lagus, Alevtina N. Danilova, Agnieszka Rewicz, Sławomir Nowak

Supplementary material 5 Cluster analysis of the similarity of populations of species of the genus *Tulipa* from East Kazakhstan according to external metric and weight indicators Data type pdf https://binary.pensoft.net/file/1221444This dataset is made available under the Open Database License (http://opendatacommons.org/licenses/odbl/1.0/). The Open Database License (ODbL) is a license agreement intended to allow users to freely share, modify, and use this Dataset while maintaining this same freedom for others, provided that the original source and author(s) are credited.Aidar A. Sumbembayev, Olga Lagus, Alevtina N. Danilova, Agnieszka Rewicz, Sławomir Nowak

Supplementary material 6 Correlation between morphometric characteristics of seeds of species of the genus *Tulipa* and environmental conditions Data type pdf Explanation note **A***Tulipapatens***B***T.altaica***C***T.biflora***D***T.uniflora***E***T.heteropetala*. https://binary.pensoft.net/file/1221445This dataset is made available under the Open Database License (http://opendatacommons.org/licenses/odbl/1.0/). The Open Database License (ODbL) is a license agreement intended to allow users to freely share, modify, and use this Dataset while maintaining this same freedom for others, provided that the original source and author(s) are credited.Aidar A. Sumbembayev, Olga Lagus, Alevtina N. Danilova, Agnieszka Rewicz, Sławomir Nowak
