# Supplementary material for: ﻿Morphometric parameters of seeds as a practical method for identifying rare species of the genus Tulipa L. (Liliaceae) from East Kazakhstan region
Source: PhytoKeys. 2025 Jan 16;251:67–86. doi: 10.3897/phytokeys.251.133890 (PMC11758096; doi:10.3897/phytokeys.251.133890)
Supplement: Supplementary material 1 — Description of the population locations of Tulipa species in East Kazakhstan region [file phytokeys-251-067_article-133890__-s001.pdf]

Supplementary Table S1. Description of the population locations of *Tulipa* species in East Kazakhstan region.

| Species, population | Location | Geographical coordinates | L* | T* | M* | R* | N* | S* | Habitat |
|---|---|---|---|---|---|---|---|---|---|
| **Family *Liliaceae* Juss.**<br>**Subfamily *Lilioideae* Eaton**<br>**Tribe *Lilieae* Ritgen**<br>**Genus *Tulipa* L.**<br>**Subgenus *Eriostemons* (Boiss.) Raamsd.**<br>**Section *Sylvestres* (Baker) Baker**<br>*Tulipa patens* C.Agardh | | | | | | | | | |
| Pop 1 | Zaisan depression, clay sedimentary ridge of Zhivan-Kara | N48.457778, E83.935556, 524 m.a.s.l. | 9 | 7 | 2 | 5 | 5 | 7 | gorge, with crushed stone of various sizes and clay formations with a small amount of humus, *Artemisia-Festuca-Poa* phytocenosis |
| Pop 2 | Southern Altai, Narym ridge, southwestern foothills, northeastern gravelly microslope, in the area of the Kurchum water crossing | N48.775, E83.472778, 420 m.a.s.l. | 8 | 6 | 4 | 7 | 6 | 6 | forb-shrub steppe area |
| Pop 3 | Southern Altai, Narym ridge, northeastern gravelly microslope, southwestern foothills | N49.206111, E84.521111, 495 m.a.s.l. | 8 | 5 | 4 | 7 | 6 | 5 | *Caragana-Agropyron* phytocenosis |
| Pop 4 | Southern Altai, Narym ridge, southwestern slope | N48.911667, E83.738889, 550 m.a.s.l. | 8 | 6 | 4 | 7 | 5 | 5 | inside bush thickets |
| Pop 5 | Southern Altai, South Altai Tarbagatai ridge, south-eastern slope | N49.095278, E86.2325, 1900 m.a.s.l. | 9 | 5 | 5 | 6,5 | 6 | 4 | forb-grass meadow |
| **Subgenus *Tulipa***<br>**Section *Kolpakowskianae* Raamsd. *ex* Zonn & Veldk.**<br>*T. altaica* Pall. *ex* Spreng. | | | | | | | | | |
| Pop 1 | Zaisan depression, clay sedimentary ridge of Zhivan-Kara, top of a leveled ridge | N48.429722, E83.978056, 511 m.a.s.l. | 9 | 7 | 3 | 8 | 4 | 6 | *Allium-Artemisia-Stipa* phytocenosis |
| Pop 2 | Zaisan depression, clay sedimentary ridge of Zhivan-Kara, peak southeastern microslope | N48.451111, E84.483889, 515 m.a.s.l. | 9 | 7 | 3 | 7 | 4 | 6 | *Artemisia–Allium* phytocenosis |
| Pop 3 | Zaisan depression, clayey sedimentary ridge of Zhivan-Kara, south-eastern slope, gorge | N48.457778, E83.935556, 524 m.a.s.l. | 9 | 7 | 3 | 7 | 5 | 5 | *Artemisia-Festuca-Poa* phytocenosis |
| Pop 4 | Zaisan depression, clay sedimentary ridge of Zhivan-Kara, south-eastern slope, deep gorge | N48.466111, E83.9125, 530 m.a.s.l. | 9 | 7 | 3 | 6 | 5 | 5 | *Artemisia-Stipa* phytocenosis |
| Pop 5 | Zaisan depression, clay sedimentary ridge of | N48.129444, E84.496389, | 9 | 7 | 4 | 6 | 5 | 5 | *Stipa* phytocenosis |

| | | | | | | | | |
|---|---|---|---|---|---|---|---|---|
| | Zhivan-Kara, northwestern slope, deep gorge | 493 m.a.s.l. | | | | | | |
| Pop 6 | Zaisan depression, Mount Kara-Biryuk, gentle slope | N47.998333, E84.817778, 520 m.a.s.l. | 9 | 7 | 3 | 5 | 4 | 5 | clayey hills |
| Pop 7 | Zaisan depression, clay ridge Kiin-Kerish | N48.125278, E84.4875, 466 m.a.s.l. | 9 | 7 | 3 | 6 | 4 | 5 | desert |
| Pop 8 | Southern Altai, southeastern spurs of the Kurchum ridge, low rocky mountains of Dalon Kara, southwestern microslope | N48.451111, E84.164444, 565 m.a.s.l. | 9 | 8 | 2 | 5 | 4 | 6 | rocky ledges |
| **Subgenus *Eriostemons* (Boiss.) Raamsd.**<br>**Section *Biflores* A.D.Hall *ex* Zonn. & Veldk.**<br><br>*T. biflora* Pall. | | | | | | | | | |
| Pop 1 | Sauro-Manrak, Manrak ridge, Kensai tract, fine-crushed southwestern slope | N47.573611, E84.185278, 800 m.a.s.l. | 9 | 7 | 3 | 5 | 4 | 5 | among sparse bushes |
| Pop 2 | Southern Altai, Narym ridge, northwestern slope, valley of river Solonechnaya | N49.206111, E84.521111, 395 m.a.s.l. | 8.5 | 6 | 4 | 5 | 4 | 5 | steppe shrub meadows |
| **Subgenus *Orithyia* (D.Don) Baker**<br>**Section *Orithyia* (D.Don) Vved.**<br>*T. uniflora* (L.) Bess. ex Baker | | | | | | | | | |
| Pop 1 | Kalba Altai, Kalba ridge, southeastern foothills | N49.490833, E82.611111, 730 m.a.s.l. | 9 | 6 | 3 | 5 | 4 | 4 | *Stipa*-*Allium* phytocenosis |
| Pop 2 | Kalba Altai, environs of the Sibin lakes, lakeside plain | N49.500833, E83.046389, 705 m.a.s.l. | 8.5 | 5 | 4 | 4 | 5 | 4 | steppe shrub meadows |
| Pop 3 | Kalba Altai, surroundings of the Sibin lakes | N49.648333, E82.2575, 583 m.a.s.l. | 9 | 5 | 4 | 4 | 5 | 4 | sparse shrub phytocenosis |
| Pop 4 | Kalba Altai, Sibinskaya depression, northwestern ridge in the Taldybulak tract | N49.9075, E82.098611, 823 m.a.s.l. | 9 | 5 | 3 | 4 | 4 | 4 | gravelly steppe |
| Pop 5 | Kalba Altai, Sibin depression, southeastern slope | N49.648333, E82.2575, 583 m.a.s.l. | 9 | 5 | 2.5 | 4 | 4 | 4 | gravelly slope of the ridge |
| Pop 6 | Kalba Altai, Sibinskaya depression, north-western gravelly slope of the ridge | N49.500278, E82.041944, 791 m.a.s.l. | 9 | 5 | 3.5 | 4 | 4 | 4 | rubble ledges |
| **Section *Orithyia* (D.Don) Vved.**<br>*T. heteropetala* Ledeb. | | | | | | | | | |
| Pop 1 | Southern Altai, Narym ridge, vicinity of the village Svinchatka, northwestern slope, Urkar river valley | N49.113333, E84.334444, 508 m.a.s.l. | 8.5 | 5 | 4 | 5 | 4.5 | 4 | Forb-grass phytocenosis |
| Pop 2 | Southern Altai, Narym ridge, Moildy tract, Moildy river valley | N49.073889, E84.189167, 517 m.a.s.l. | 9 | 5.5 | 3.5 | 5 | 3.5 | 4.5 | Clayey steppe ledges |
| Pop 3 | Southern Altai, Azutau ridge, Mount Mramornaya | N48.373056, E85.516111, 886 m.a.s.l. | 9 | 6 | 3 | 5 | 3 | 4 | Rocky slopes, in communities with *Festuca* |

| | | | | | | | | | |
|---|---|---|---|---|---|---|---|---|---|
| Pop 4 | Southwestern Altai, Kholzun ridge, southeastern slope, eastern microslope | N 50.321667, E 84.25. 920 m.a.s.l.. | 9 | 4 | 3.5 | 4.5 | 3 | 4 | Steppe rocky slopes |
| Pop 5 | Southwestern Altai, Kholzun ridge, upper limit of the southwestern peak | N50.081944, E84.511389, 924 m.a.s.l. | 8.5 | 4 | 4.5 | 4 | 5 | 3 | *Hedysarum* microphytocenosis. |
| Pop 6 | Southwestern Altai, Kholzun ridge, southeastern slope, near the village of Maleevka. | N49.971283, E84.262894 845 m.a.s.l. | 9 | 4.5 | 3 | 4 | 3 | 4 | On placers of crushed stone |
| Pop 7 | Southern Altai, Bukhtarma Mountains, Shybyn-Bulak tract, environs of the village of Katon-Karagai | N49.189444, E85.5175, 895 m.a.s.l. | 9 | 5 | 4 | 4.5 | 3 | 3.5 | Steppe clay slopes |
| Pop 8 | Zaisan basin, valley of the Black Irtysh river | N48.451111, E83.978056, 483 m.a.s.l. | 9 | 6 | 3.5 | 5 | 3.5 | 5 | Steppe meadows |
| Pop 9 | Zaisan depression, Ak-Seir tract, disturbed areas of the northwestern microslope | N 48.466111, E 83.9125, 530 m.a.s.l. | 9 | 6 | 3 | 4.5 | 3 | 4.5 | clayey gravelly ledges |
| Pop 10 | Zaisan depression, semi-desert | N 48.009444, E 85.341944, 408 m.a.s.l. | 9 | 7 | 2 | 5 | 2.5 | 6 | On clay hills |

Verbal definitions of the scales of Ellenberg indicator values.
**L – Light** (scale 1–9)
1 – deep shade plant, occurring where the incident diffuse radiation is less than 1% of that in an open area, rarely at more than 30%
2 – between 1 and 3
3 – shade plant, usually occurring where the incident diffuse radiation is less than 5% of that in an open area, but also at sunnier sites
4 – between 3 and 5
5 – semi-shade plant, only exceptionally occurring in full light, but usually at more than 10% of the diffuse radiation incident in an open area
6 – between 5 and 7; rarely at less than 20% of diffuse radiation incident in an open area
7 – half-light plant, mostly occurring at full light, but also in the shade up to about 30% of diffuse radiation incident in an open area
8 – light plant, only exceptionally occurring at less than 40% of diffuse radiation incident in an open area
9 – full light plant, occurring only in fully irradiated places, not at less than 50% of diffuse radiation incident in an open area
**T – Temperature** (scale 1–9)
1 – cold indicator, only in high mountain areas, i.e. the alpine and nival belts
2 – between 1 and 3 (many alpine species)
3 – cool indicator, mainly in subalpine areas
4 – between 3 and 5 (especially high montane and montane species)
5 – moderate heat indicator, from lowland to montane belt, mainly in submontane-temperate areas
6 – between 5 and 7 (lowland and colline species)
7 – heat indicator, occurring in relatively warm lowlands
8 – between 7 and 9
9 – extreme heat indicator
**M – Moisture** (scale 1–12)
1 – strong drought indicator, viable at sites that frequently dry out and confined to dry soils
2 – between 1 and 3
3 – missing on damp soil
4 – between 3 and 5
5 – indicator of fresh soils, focus on soils of average moisture, missing on wet soils and on soils that frequently dry out
6 – between 5 and 7
7 – humidity indicator, focus on well moistened, but not wet soils
8 – between 7 and 9
9 – wetness indicator, focus on often soaked, poorly aerated soils
10 – aquatic plant that survives long periods without soil flooding

11 – aquatic plant rooted under water, but at least temporarily with leaves above the surface, or a plant floating on the water surface
12 – permanently or almost permanently submerged aquatic plant
**R – Reaction** (scale 1–9)
1 – indicator of strong acidity, never occurring in slightly acidic to alkaline conditions
2 – between 1 and 3
3 – acidity indicator, occurring mainly in acidic conditions, exceptionally in neutral conditions
4 – between 3 and 5
5 – indicator of moderate acidity, occurring rarely in strongly acidic as well as in neutral to alkaline conditions
6 – between 5 and 7
7 – indicator of slightly acidic to slightly basic conditions, never occurring in very acidic conditions
8 – between 7 and 9, occurring mostly in calcium-rich conditions
9 – base and lime indicator, always occurring in calcium-rich conditions
**N – Nutrients** (scale 1–9)
1 – occurring at nutrient-poorest sites
2 – between 1 and 3
3 – occurring at nutrient-poor sites more frequently than at average sites and exceptionally at rich sites
4 – between 3 and 5
5 – occurring at moderately nutrient-rich sites, and less frequently at poor and rich sites
6 – between 5 and 7
7 – occurring at nutrient-rich sites more often than at average sites and only exceptionally at poor sites
8 – pronounced nutrient indicator
9 – concentrated at very nutrient-rich sites
**S – Salinity** (scale 0–9)
0 – not salt tolerant, glycophyte
1 – salt tolerant, mostly on low-salt to salt-free soils, but occasionally on slightly salty soils
2 – oligohaline, often on soils with very low salt content
3 – -mesohaline, mostly on soils with low salt content
4 – /-mesohaline, mostly on soils with low to moderate salt content
5 – -mesohaline, mostly on soils with a moderate salt content
6 – -meso/polyhaline, on soils with moderate to high salt content
7 – polyhaline, on soils with a high salt content
8 – euhaline, on soils with a very high salt content
9 – euhaline to hypersaline, on soils with a very high and in dry periods extremely high salt content
