# Supplementary material for: ﻿Morphometric parameters of seeds as a practical method for identifying rare species of the genus Tulipa L. (Liliaceae) from East Kazakhstan region
Source: PhytoKeys. 2025 Jan 16;251:67–86. doi: 10.3897/phytokeys.251.133890 (PMC11758096; doi:10.3897/phytokeys.251.133890)
Supplement: Supplementary material 2 — Morphometric characteristics of Tulipa seeds from different growing areas [file phytokeys-251-067_article-133890__-s002.pdf]

Supplementary Table S2. Morphometric characteristics of *Tulipa* seeds from different growing areas.

Species, population	Seed length, mm		Seed width, mm		Seed thickness, mm		Weight of 1000 seeds, g.
	*M±m<br>Min–Max	Cv, %	M±m<br>Min–Max	Cv, %	M±m<br>Min–Max	Cv, %	
T. patens, Pop 1	4.573±0.124<br>3.95 – 4.93	5.65	3.298±0.123<br>2.77 – 3.80	7.59	0.247±0.01<br>0.20 – 0.28	8.41	1.87
T. patens, Pop 2	4.424±0.138<br>3.93 – 5.04	5.88	3.165±0.129<br>2.82 – 3.76	7.42	0.286±0.023<br>0.21 – 0.40	16.35	2.08
T. patens, Pop 3	4.617±0.162<br>4.05 – 5.58	7.35	3.099±0.115<br>2.83 – 3.51	7.73	0.276±0.022<br>0.21 – 0.36	15.25	2.15
T. patens, Pop 4	4.839±0.158<br>4.25 – 5.33	7.02	3.293±0.189<br>2.88 – 4.25	10.48	0.298±0.032<br>0.22 – 0.41	20.46	2.11
T. patens, Pop 5	3.755±0.181<br>3.21 – 4.69	9.44	2.942±0.305<br>2.38 – 4.01	20.26	0.959±0.066<br>0.77 – 1.16	13.42	2.30
T. altaica, Pop 1	5.331±0.179<br>4.71 – 6.48	7.19	4.237±0.213<br>3.35 – 5.04	10.76	0.395±0.053<br>0.28 – 0.57	24.7	3.35
T. altaica, Pop 2	6.223±0.323<br>5.38 – 7.15	9.44	4.864±0.175<br>4.27 – 5.28	6.54	0.346±0.024<br>0.26 – 0.42	12.96	4.75
T. altaica, Pop 3	5.975±0.278<br>5.01 – 6.94	9.96	4.895±0.25<br>3.86 – 5.67	9.3	0.341±0.02<br>0.27 – 0.45	13.07	4.88
T. altaica, Pop 4	5.917±0.162<br>5.23 – 6.69	5.86	4.718±0.166<br>4.19 – 5.16	6.39	0.375±0.029<br>0.28 – 0.48	14.31	4.64
T. altaica, Pop 5	5.861±0.196<br>5.05 – 6.31	6.09	4.504±0.212<br>3.86 – 5.25	10.10	0.346±0.035<br>0.25 – 0.51	18.85	3.756
T. altaica, Pop 6	5.874±0.24<br>5.35 – 6.83	7.71	4.697±0.22<br>3.88 – 5.82	10.04	0.366±0.036<br>0.24 – 0.48	21.32	4.01
T. altaica, Pop 7	5.363±0.153<br>4.91 – 5.93	5.39	5.338±0.205<br>4.71 – 6.33	8.25	0.398±0.032<br>0.29 – 0.60	19.76	3.58
T. altaica, Pop 8	5.841±0.225<br>5.02 – 6.66	7.28	4.601±0.171<br>3.81 – 4.98	7.93	0.346±0.027<br>0.24 – 0.46	16.71	4.24
T. biflora, Pop 1	4.583±0.129<br>4.13 – 4.95	6.04	3.618±0.168<br>3.09 – 4.26	9.98	0.536±0.045<br>0.39 – 0.74	18.2	3.23
T. biflora, Pop 2	4.789±0.192<br>4.01 – 5.49	8.58	3.244±0.201<br>2.74 – 4.11	13.21	0.398±0.039<br>0.31 – 0.67	21.06	2.96
T. uniflora, Pop 1	4.396±0.166<br>3.80 – 5.19	8.10	1.937±0.09<br>1.60 – 2.48	9.96	1.02±0.072<br>0.82 – 1.31	12.82	3.764
T. uniflora, Pop 2	4.586±0.152<br>4.14 – 5.42	7.07	2.135±0.099<br>1.87 – 2.77	9.97	1.205±0.068<br>0.95 – 1.47	12.01	4.37
T. uniflora, Pop 3	4.855±0.215<br>4.16 – 5.79	9.49	2.224±0.306<br>1.79 – 4.67	29.43	1.257±0.075<br>0.98 – 1.57	12.89	5.28
T. uniflora, Pop 4	3.956±0.221<br>3.21 – 5.23	11.92	1.576±0.102<br>1.29 – 2.03	11.79	0.944±0.104<br>0.74 – 1.36	20.14	4.02
T. uniflora, Pop 5	3.871±0.173<br>3.06 – 4.44	8.76	1.801±0.109<br>1.49 – 2.26	13.01	1.078±0.096<br>0.85 – 1.47	16.23	3.282
T. uniflora, Pop 6	3.654±0.247<br>3.02 – 4.48	12.30	1.702±0.139<br>1.35 – 2.32	16.01	1.105±0.062<br>0.88 – 1.29	11.47	3.491
T. heteropetala, Pop 1	3.494±0.158<br>3.01 – 3.92	8.24	2.319±0.149<br>2.11 – 3.13	11.74	0.631±0.068<br>0.45 – 0.86	19.49	2.214

T. heteropetala, Pop 2	3.758±0.216<br>3.28 – 4.97	10.45	2.403±0.122<br>2.06 – 2.76	9.29	0.705±0.048<br>0.54 – 0.83	12.54	2.75
T. heteropetala, Pop 3	3.777±0.186<br>3.12 – 4.32	8.97	2.725±0.161<br>2.12 – 3.26	11.17	0.735±0.074<br>0.56 – 0.96	18.35	2.9
T. heteropetala, Pop 4	4.329±0.101<br>4.01 – 4.71	4.59	2.915±0.122<br>2.44 – 3.26	7.62	0.603±0.076<br>0.41 – 0.91	25.37	3.03
T. heteropetala, Pop 5	3.56±0.115<br>3.06 – 3.97	6.33	2.406±0.199<br>1.76 – 2.92	15.01	0.84±0.106<br>0.63 – 1.29	22.93	2.74
T. heteropetala, Pop 6	4.23±0.141<br>3.69 – 4.68	6.32	2.974±0.192<br>2.50 – 3.16	11.73	0.574±0.075<br>0.41 – 0.84	23.8	2.87
T. heteropetala, Pop 7	3.728±0.108<br>3.40 – 3.98	5.47	2.472±0.134<br>2.09 – 3.01	10.27	0.554±0.035<br>0.43 – 0.75	12.84	2.616
T. heteropetala, Pop 8	4.424±0.169<br>3.93 – 4.98	6.98	2.833±0.128<br>2.48 – 3.23	8.51	0.559±0.051<br>0.35 – 0.78	19.49	3.22
T. heteropetala, Pop 9	3.376±0.115<br>3.01 – 3.84	6.46	2.253±0.136<br>1.80 – 2.75	11.01	0.563±0.077<br>0.35 –0.84	26.74	2.193
T. heteropetala, Pop 10	4.471±0.199<br>3.83 – 4.89	9.53	3.068±0.24<br>2.48 – 3.84	14.24	0.567±0.065<br>0.39 – 0.83	20.79	3.068
* M – the average value of the metric feature, m – allowable limits, min -max – minimum and maximum feature values, Cv – coefficient of variation of a feature.							
