# Supplementary material for: ﻿Morphometric parameters of seeds as a practical method for identifying rare species of the genus Tulipa L. (Liliaceae) from East Kazakhstan region
Source: PhytoKeys. 2025 Jan 16;251:67–86. doi: 10.3897/phytokeys.251.133890 (PMC11758096; doi:10.3897/phytokeys.251.133890)
Supplement: Supplementary material 3 — Indicators of morphological characteristics of seeds of species of the genus Tulipa in East Kazakhstan region [file phytokeys-251-067_article-133890__-s003.pdf]

Supplementary Table S3. Indicators of morphological characteristics of seeds of species of the genus *Tulipa* in East Kazakhstan region.

Morphological characteristics of seeds	Species name				
	Tulipa patens	*Tulipa altaica*	*Tulipa biflora*	*Tulipa uniflora*	*Tulipa heteropetala*
Dimensions, shape	Small (*parvum**), flattened (*applanatum*)	Large (*magnum*), flat (*complanatum*)	Small (*parvum*), flat (*complanatum*)	Small (*parvum*), slightly flattened (*applanatum*), noticeably voluminous in the basal part	Small (*parvum*), flattened (*applanatum*), slightly skewed in plane
Length, mm	3.21 – 5.58 (avg. 4.442)	4.71 – 7.15 (avg. 5.798)	4.01 – 5.49 (avg. 4.686)	3.02 – 5.79 (avg. 4.220)	3.01 – 4.97 (avg. 3.781)
Width, mm	2.38 – 4.25 (avg. 3.157)	3.35 – 6.33 (avg. 4.732)	2.74 – 4.26 (avg. 3.462)	1.29 – 2.77 (avg. 2.088)	1.76 – 3.84 (avg. 2.558)
Thickness, mm	0.20 – 0.41 (avg. 0.413)	0.24 – 0.6 (avg. 0.364)	0.31 – 0.74 (avg. 0.497)	0.74 – 1.57 (avg. 0.944)	0.35 – 1.29 (avg. 0.588)
Weight 1000 pcs., g.	2.175	4.151	3.230	3.967	2.635
Seed color range	Matte (*opacum*), slightly lighter around the edges. Variations of brown (*brunneum*)	From orange–brown (*aurantiaco-brunneum*) to brown (*brunneum*), with a slight glossy tint (*nitidum*), unicolorous	Light gloss (*nitidum*), unicolorous. From orange–brown (*aurantiaco-brunneum*) to red–brown (*rubeolo-fuscum*)	Matte (*opacum*), rarely with a slight gloss (*nitidum*), unicolorous. From gray–violet (*griseo-violaceum*) to dark brown (*fusco-brunneum*)	Matte (*opacum*), from orange–brown (*aurantiaco-brunneum*) to reddish-brown (*rubeolo-fuscum*) with a slight glossy tint (*nitidum*)
Seed shape	Vaguely triangular (*triangulare*), slightly convex to one side, looks	Triangular (*triangulare*), convex to one side, with smoothed corners, with the micropylar end noticeably elongated. Flat	Wedge-shaped (*cuneiforme*), and in the chalazal part the edge of the seed is evenly rounded. Sometimes the back of the seed is convex to one side. Flat	Curved navicular (*curvatum cymbiforme*), with concave side, sometimes	Curved wedge-shaped (*curvatum cuneiforme*), sometimes

	like a skewed sector of a circle			biconcave, the hilum and chalaza are always turned toward the raphe, thickened in the basal part.	triangular (*triangulare*) with rounded corners, curved in the back, partially skewed in plane. Flat
Testa surface	Slightly rough (*scabrum*), without pubescence. Along the longitudinal edge of the seed there is a noticeable thickening in the form of a curved border.	Thin (*tenue*), membranous in places (*paleaceum*), rough (*scabrum*), with small veins, naked (*glabrum*). The lateral edges of the seed are thickened, slightly curved upward, rather smooth, sometimes wrinkled	Rough (*scabrum*), with small furrows, rarely wrinkled (*rugosum*), naked (*glabrum*). The lateral edge of the seed is curved upward, wavy, longitudinally veined	Quite dense, abundantly wrinkled (*rugosum*), sometimes longitudinally grooved (*longitudinaliter-sulcatum*), naked (*glabrum*), rough (*scabrum*), sometimes finely tuberculate (*tuberculatum*). The lateral edge of the seed is slightly wavy, slightly curved upward	Thin dough (*tenue*), wrinkled (*rugosum*), covered with abundant tubercles (*tuberculatum*), furrowed in places (*sulcatum*). Testa surface is naked (*glabrum*), membranous (*paleaceum*). The lateral edge of the seed is slightly curved upward, slightly wavy, naked.
Micropyle condition (*micropyle*)	Overgrown, located in the narrow elongated tip of the seed	Clearly, visible, it is shaped like a crescent, completely overgrown. The micropylar end tapers sharply	Overgrown, round or rarely concave-round in shape, located in the sharply tapered tip of the seed	Heavily overgrown with internal integuments, evenly rounded, located in the	Overgrown, located in the seed tip inclined toward the raphe. The micropylar

				curved tip of the seed	entrance is clearly visible
Features of the chalazal end (*chalaza*)	Strongly thickened, sometimes abundantly covered with tubercles	Strongly thickened, quite smooth	Thickened, quite wide in the transverse part, proportionally rounded, sometimes slightly sloping toward the raphe. The surface of the chalaza is bare, slightly rough	Thickened, sometimes concave, glabrous, abundantly covered with transverse wavy grooves	Noticeably enlarged, without pubescence, has a more wrinkled surface
Type and shape of seed hilum (*hilum*)	Small (*parvum*), linear (*lineare*), slightly protruding (*exsertum*), longitudinally slit-shaped (*longitudinaliter rimiforme*), located in the basal part	Small (*parvum*), linear (*lineare*), longitudinally slit-shaped (*longitudinaliter rimiforme*), slightly pressed in (*impressum*), located in the basal part	Small (*parvum*), linear type (*lineare*), slightly concave (*impressum*), longitudinally slit-shaped (*longitudinaliter rimiforme*), located in the basal part	Small (*parvum*), slightly concave (*impressum*), surrounded by a small cushion (*valliculo cinctum*), ellipsoidal shape (*ellipsoideum*), located in the basal part	Small (*parvum*), linear type (*lineare*), located in the basal part, slightly concave (*impressum*), ellipsoidal (*ellipsoideum*)
Structure and surface of raphe (*raphe*)	Short (*brevis*), wide (*lata*), smooth, located on a flat side edge	Short (*brevis*), barely noticeable, narrow (*angusta*), smooth	Short (*brevis*), located on the side edge, clearly visible, wide (*lata*), slightly rough	Short (*brevis*), highly visible, wide (*lata*), has a wavy shape, rough	Short (*brevis*), rectilinear shape, sometimes wide (*lata*), has a grooved, smooth surface in places
Endosperm (*endospermium*)	Soft (*molle*), well developed, fills a large space, clear	Soft (*molle*), developed, transparent, completely surrounds the embryo	Soft (*molle*), clear, well developed, occupies the main space in the seed	Soft (*molle*), developed, rarely underdeveloped, transparent, occupies a	Soft (*molle*), developed, transparent, occupies a large space of the seed

				relatively small space in the seed	
Embryo (*embryo*)	Small (*parvus*), has a white color, does not contain chlorophyll, straight form (*rectus*), located close to the micropyle, completely surrounded by endosperm	Quite large, has a white color, does not contain chlorophyll, has a rectilinear shape (*rectus*), very rarely slightly curved (*curvatus*), completely surrounded by endosperm, located perpendicular to the micropyle and noticeably distant from the chalaza, and the end of the embryo, close to the micropyle, is thinner than the other	Small (*parvus*), has a white color, does not contain chlorophyll. linear shape (*linearis*), always close to the micropyle and distant from the chalazal end, often curved toward the raphe, but completely surrounded by endosperm, slightly narrowed in the area of the micropyle	Small (*parvus*), has a light milky color, does not contain chlorophyll, strongly curved (*curvatus*), completely surrounded by endosperm, located close to the micropyle	Small (*parvus*), has a white color, does not contain chlorophyll, linear shape (*linearis*), located close to the micropyle, completely surrounded by endosperm
Shape of individuals testa cells	Elongated and more or less rectangular at the end	Elongated and rectangular at the end/and more or less irregular cells at the edges of the seed	Elongated and rectangular at the end/and a lot of irregular cells at the edges of the seed	Elongated and more or less rectangular at the end	Elongated and more or less rounded at the end/more or less rectangular cells at the edges of the seed
Anticlinal wall	Straight	Straight	Straight	Straight	Straight
Periclinal wall	Granular	Smooth	Smooth	Smooth	Smooth
* Latin terms in brackets					
