# Supplementary material for: ﻿Morphometric parameters of seeds as a practical method for identifying rare species of the genus Tulipa L. (Liliaceae) from East Kazakhstan region
Source: PhytoKeys. 2025 Jan 16;251:67–86. doi: 10.3897/phytokeys.251.133890 (PMC11758096; doi:10.3897/phytokeys.251.133890)
Supplement: Supplementary material 4 — Seed coat micromorphology of Tulipa species, seen under Scaning Electron Microscope [file phytokeys-251-067_article-133890__-s004.pdf]

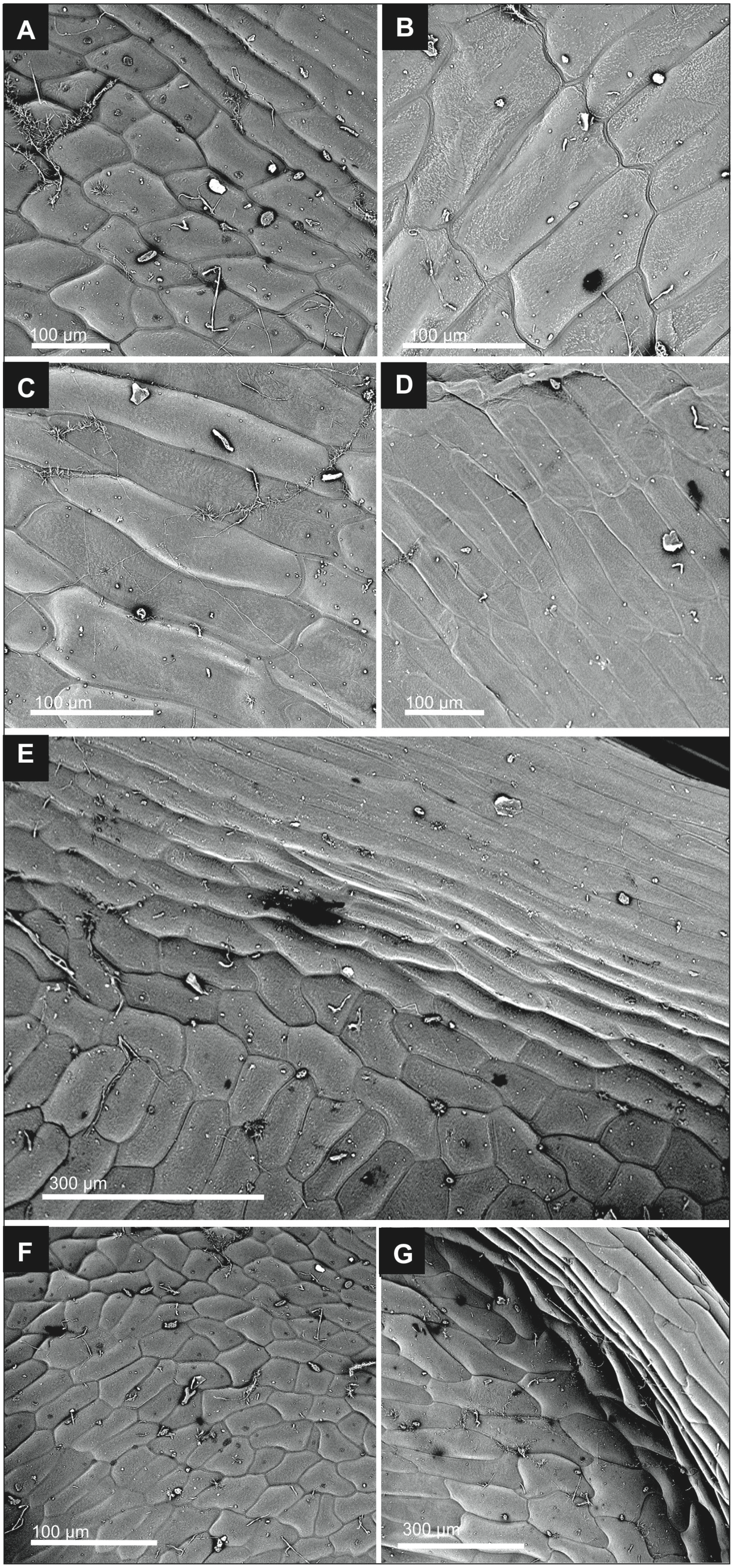

Supplementary Fig. S1. Seed coat micromorphology of *Tulipa altaica*: A-D – shape of the seed coat cells, E-G – general view.

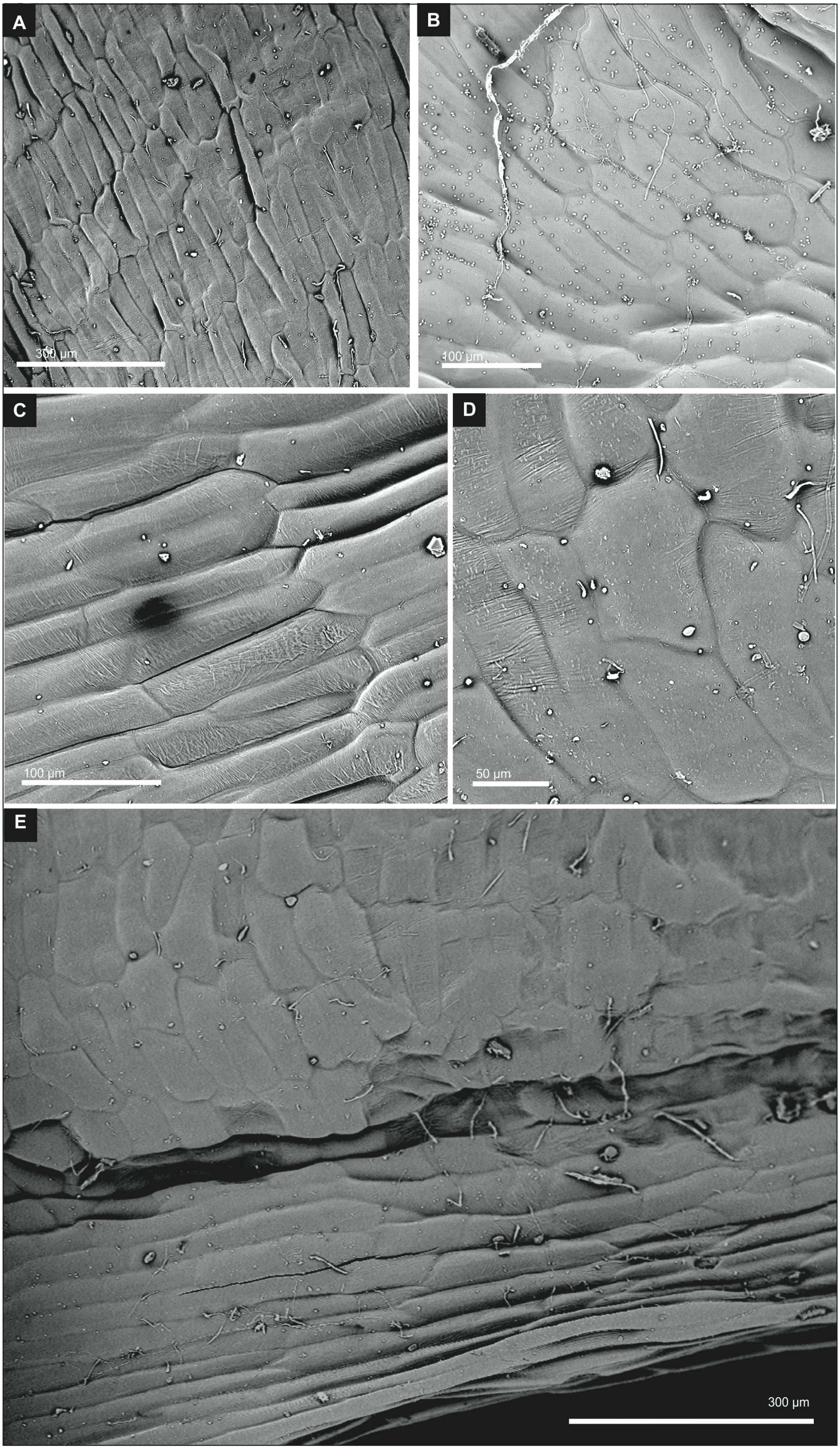

Supplementary Fig. S2. Seed coat micromorphology of *Tulipa biflora*: A-D – shape of the seed coat cells, E – general view.

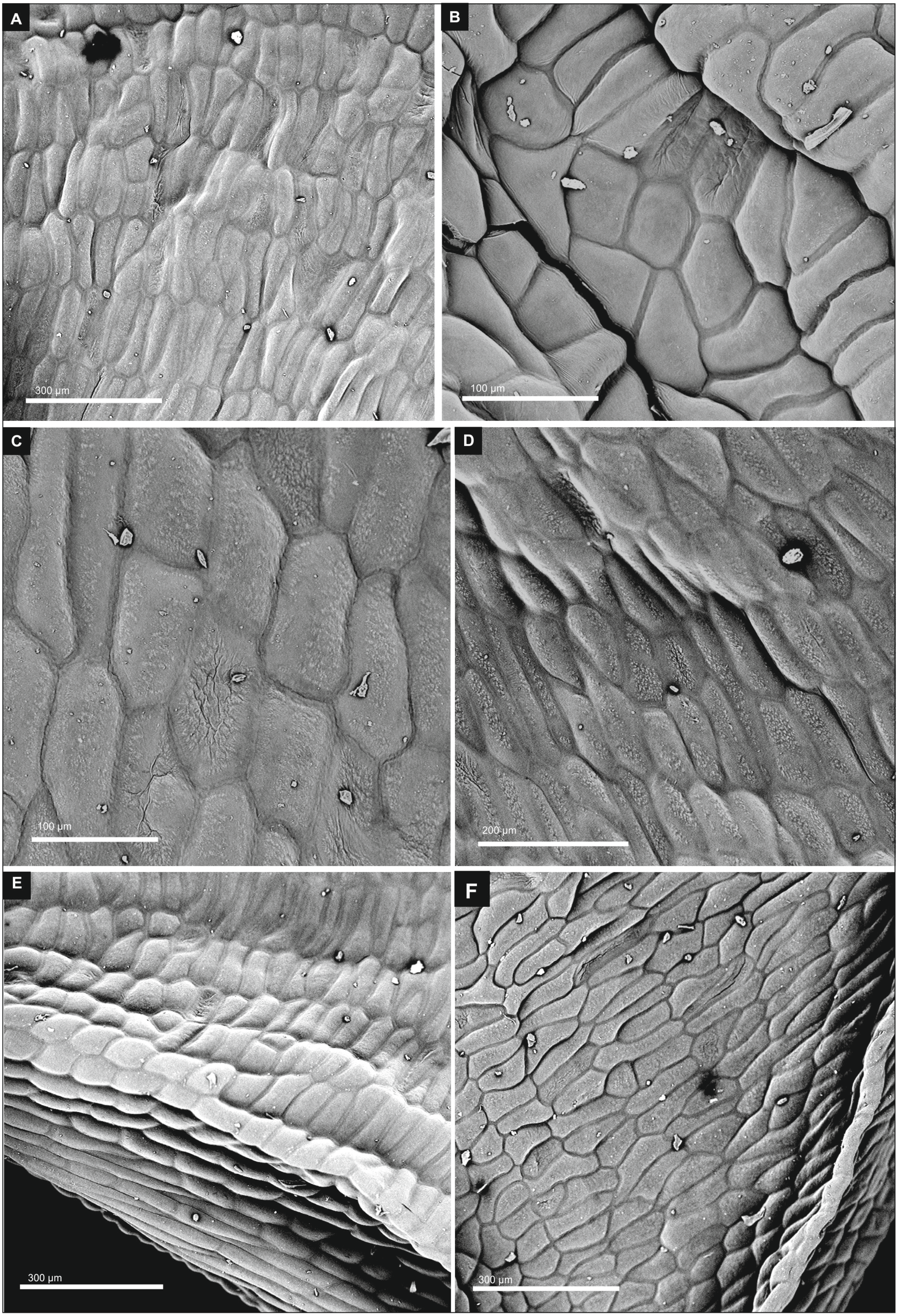

Supplementary Fig. S3. Seed coat micromorphology of *Tulipa heteropetala*: A-D – shape of the seed coat cells, E-F – general view.

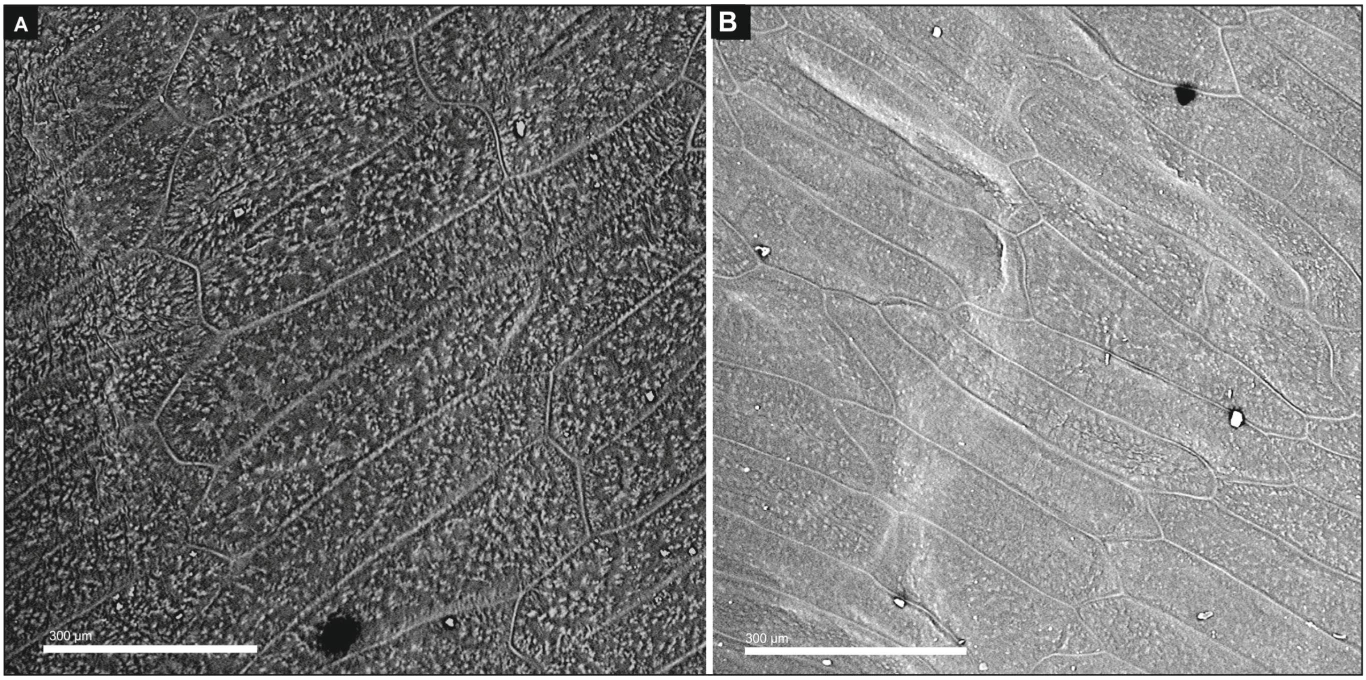

Supplementary Fig. S4. Seed coat micromorphology of *Tulipa patens*: A-B – shape of the seed coat cells.

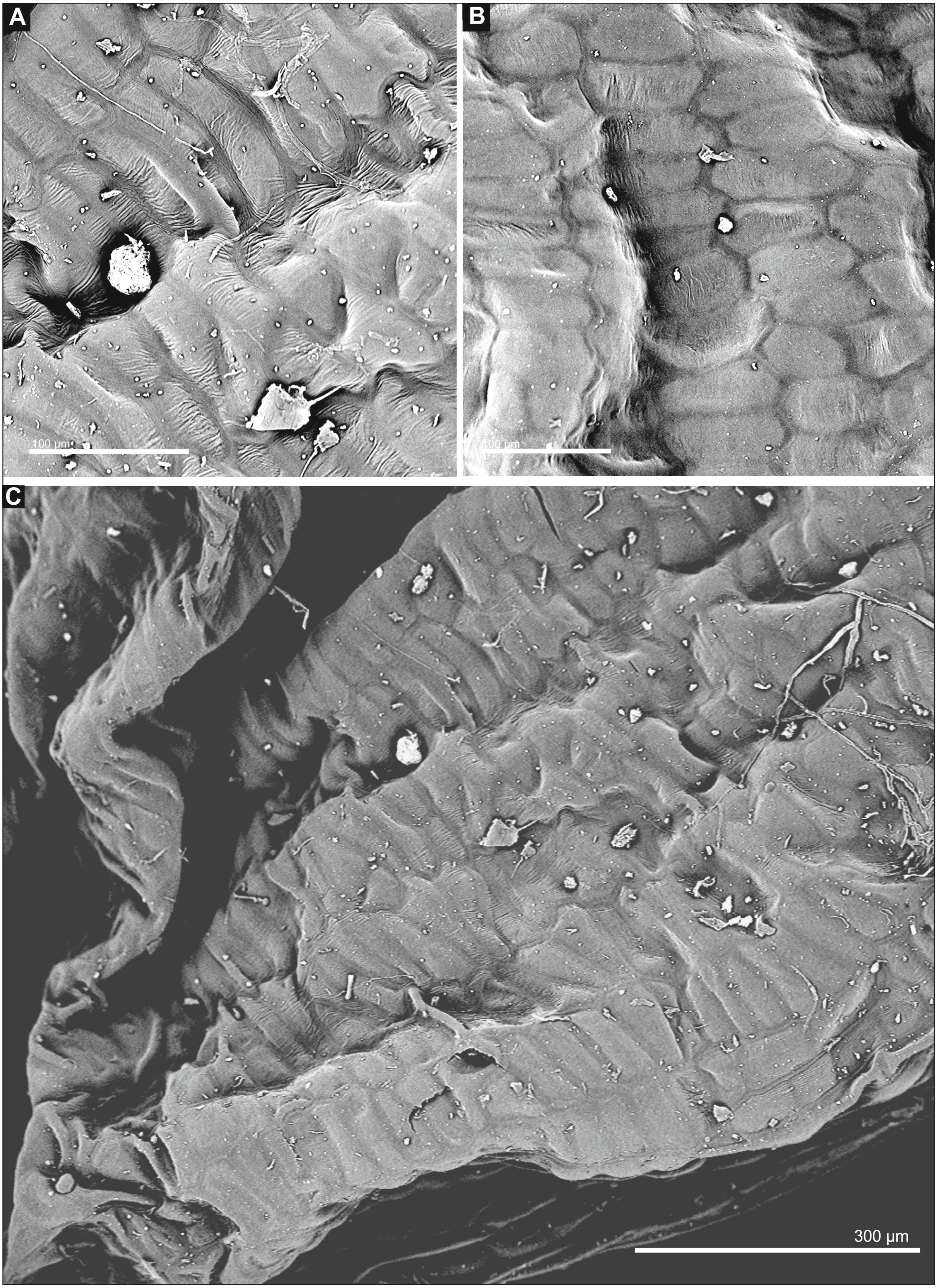

Supplementary Fig. S5. Seed coat micromorphology of *Tulipa uniflora:* A-B – shape of the seed coat cells, C – general view.
