# Supplementary material for: ﻿Morphometric parameters of seeds as a practical method for identifying rare species of the genus Tulipa L. (Liliaceae) from East Kazakhstan region
Source: PhytoKeys. 2025 Jan 16;251:67–86. doi: 10.3897/phytokeys.251.133890 (PMC11758096; doi:10.3897/phytokeys.251.133890)
Supplement: Supplementary material 5 — Cluster analysis of the similarity of populations of species of the genus Tulipa from East Kazakhstan according to external metric and weight indicators [file phytokeys-251-067_article-133890__-s005.pdf]

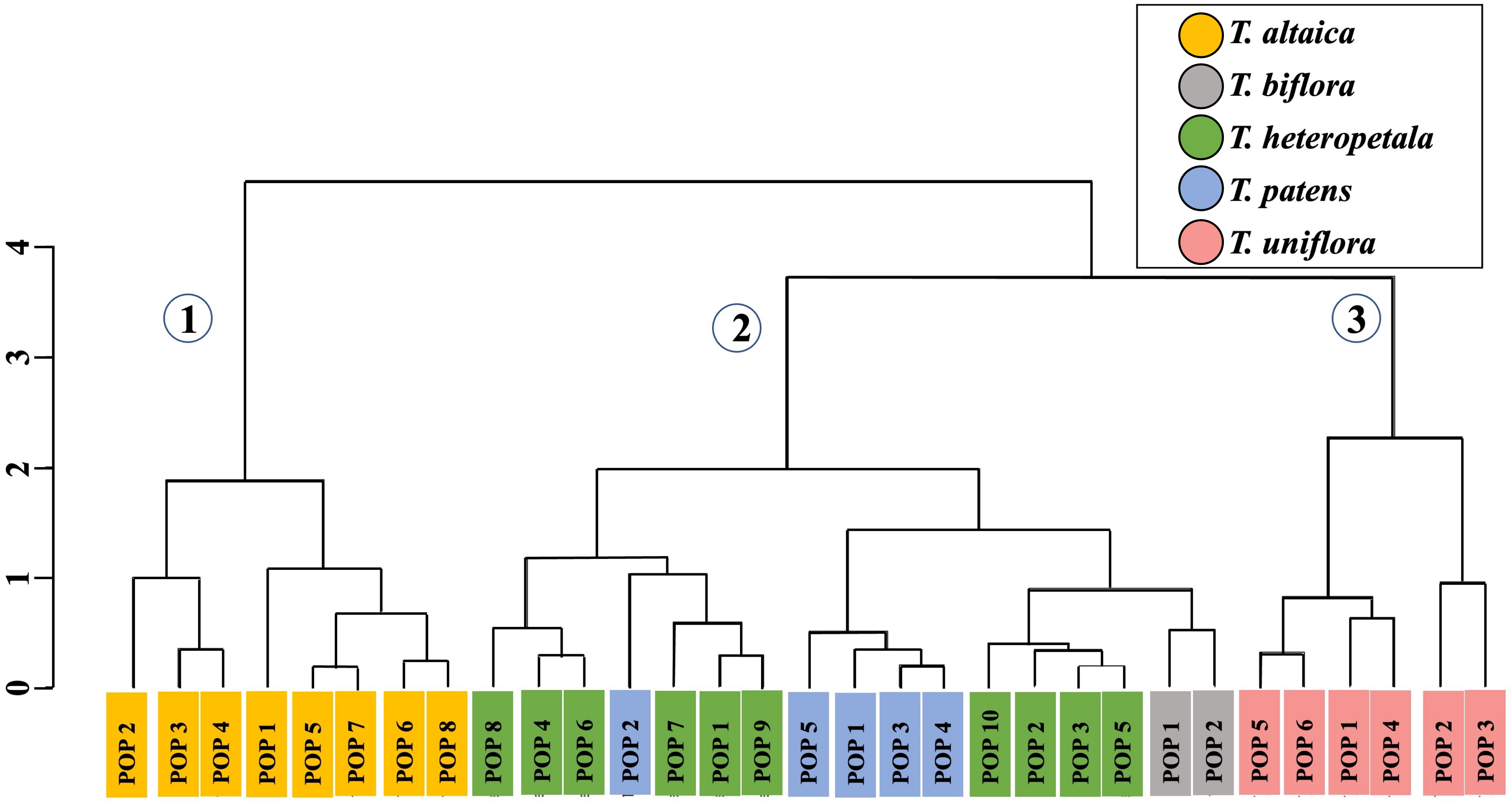

Supplementary Fig. S6. Cluster analysis of the similarity of populations of species of the genus *Tulipa* from East Kazakhstan according to external metric and weight indicators.
