# Supplementary material for: ﻿Morphometric parameters of seeds as a practical method for identifying rare species of the genus Tulipa L. (Liliaceae) from East Kazakhstan region
Source: PhytoKeys. 2025 Jan 16;251:67–86. doi: 10.3897/phytokeys.251.133890 (PMC11758096; doi:10.3897/phytokeys.251.133890)
Supplement: Supplementary material 6 — Correlation between morphometric characteristics of seeds of species of the genus Tulipa and environmental conditions [file phytokeys-251-067_article-133890__-s006.pdf]

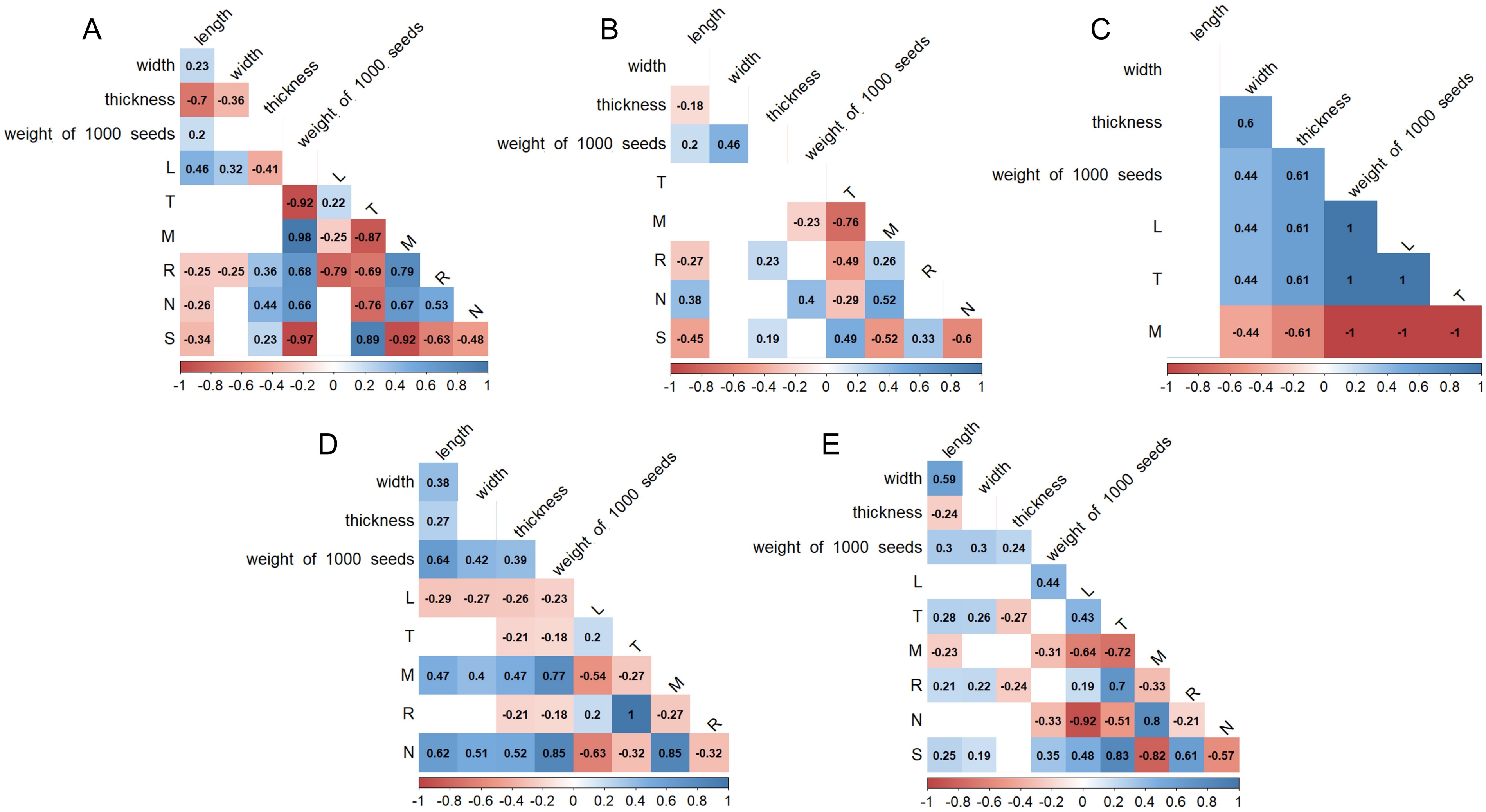

Supplementary Fig. S7. Correlation between morphometric characteristics of seeds of species of the genus *Tulipa* and environmental conditions: A – *T. patens*, B – *T. altaica*, C – *T. biflora*, D – *T. uniflora*, E -–*T. heteropetala*.
